# Inhibition of Host Gene Expression by KSHV: Sabotaging mRNA Stability and Nuclear Export

**DOI:** 10.3389/fcimb.2021.648055

**Published:** 2021-04-09

**Authors:** Carissa Ikka Pardamean, Ting-Ting Wu

**Affiliations:** Department of Molecular and Medical Pharmacology, University of California, Los Angeles, CA, United States

**Keywords:** gammaherpesvirus, Kaposi sarcoma-associated herpesvirus (KSHV), host mRNA nuclear export, mRNA stability, host gene expression inhibition, virus host interaction

## Abstract

Viruses are known for their ability to alter host gene expression. Kaposi sarcoma-associated herpesvirus has two proteins that obstruct host gene expression. KSHV SOX, encoded by the open reading frame 37 (ORF37), induces a widespread cytoplasmic mRNA degradation and a block on mRNA nuclear export. The other KSHV protein, encoded by the open reading frame 10 (ORF10), was recently identified to inhibit host gene expression through its direct function on the cellular mRNA export pathway. In this review, we summarize the studies on both SOX and ORF10 in efforts to elucidate their mechanisms. We also discuss how the findings based on a closely related rodent virus, murine gammaherpesvirus-68 (MHV-68), complement the KSHV findings to decipher the role of these two proteins in viral pathogenesis.

## Introduction

Kaposi sarcoma-associated herpesvirus (KSHV), or the human herpesvirus-8 (HHV-8), belongs to the gamma subfamily of herpesviruses. KSHV is associated with malignancies and life-threatening diseases (Chang et al., 1994; Soulier et al., 1995; Brambilla et al., 1996; Cesarman et al., 1996; Corbellino et al., 1996a; Corbellino et al., 1996b; Dittmer and Damania, 2013; Goncalves et al., 2017), including Kaposi sarcoma, primary effusion lymphoma, multicentric Castleman’s disease, and KSHV inflammatory cytokine syndrome. KSHV has two distinct phases in its life cycle: latency and lytic replication. Latency is considered to be immunologically silent with few viral genes expressed and no virion production. During lytic replication, over 80 viral genes are expressed in a cascading order, resulting in the assembly and release of infectious particles, generating new infections to replenish the pool of latently infected cells.

Restricted host range limits most KSHV studies to molecular biology experiments. Murine gammaherpesvirus-68 (MHV-68) is closely related to human gammaherpesviruses (Virgin et al., 1997; Barton et al., 2011). Like KSHV, MHV-68 infection in mice leads to acute lytic viral replication followed by the establishment of latency in B-cells. 63 out of the 80 MHV-68 open reading frames (ORFs) share sequence homology with KSHV ORFs with 10-60% shared sequence identity (Virgin et al., 1997). Unlike KSHV, MHV-68 *de novo* infection readily leads to lytic replication. Therefore, MHV-68 provides a valuable model for *in vitro* and *in vivo* functional studies of KSHV viral protein homologs.

**Figure 1:**
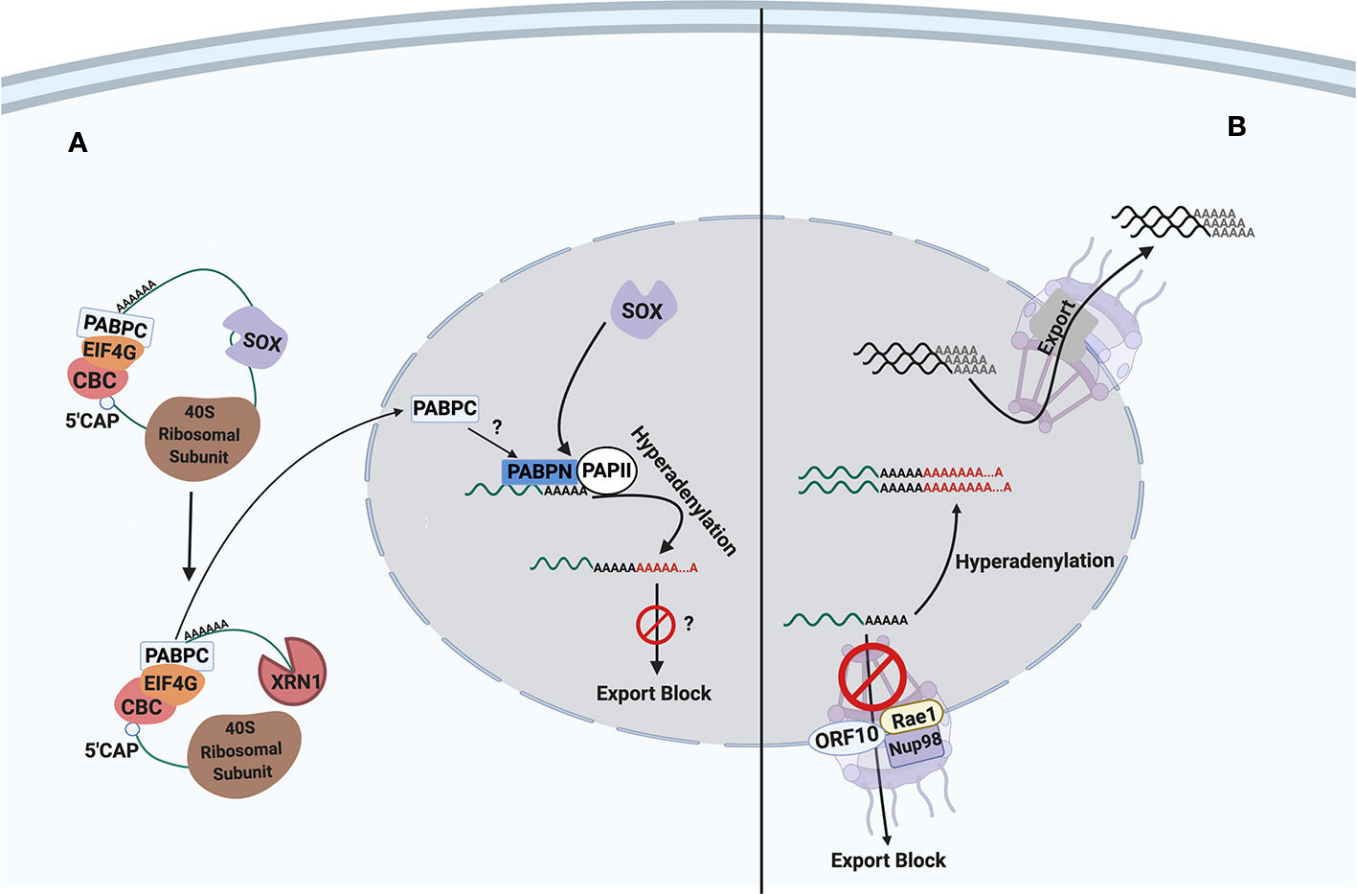
The inhibitory mechanisms of SOX and ORF10 proteins on host gene expression. SOX protein targets transcripts for degradation, affecting both the host and viral transcripts. On the other hand, ORF10 acts solely on cellular transcripts for export inhibition. Interestingly, both SOX and ORF10 cause the hyperadenylation of transcripts within the nucleus. **(A)** Cytoplasmic SOX protein, as encoded by ORF37, causes host shut off through its endonuclease activity. The viral endonucleolytic action on host transcript then triggers the cellular exonuclease, Xrn1, to complete transcript degradation. **(B)** At the nuclear envelope, ORF10 inhibits only the export of host transcripts by forming a complex with cellular export factor Rae1 and its partnering nucleoporin, Nup98. The hijacking of Rae1-Nup98 leads to nuclear retention of mRNAs. CBC, cap-binding complex; PABPC, cytoplasmic poly(A)-binding protein; PABPN, nuclear poly(A)-binding protein; PAPII, poly(A) polymerase II. Question marks indicate potential or hypothetical mechanisms and effects within the model.

Viruses strategically exploit various cellular mechanisms to down-regulate host gene expression for their own benefit. The best studied KSHV protein to inhibit host gene expression is the shutoff and exonuclease (SOX) protein encoded by KSHV ORF37 (Glaunsinger and Ganem, 2004), which accelerates cytoplasmic mRNA degradation. Another common viral strategy, frequently employed by RNA viruses, targets mRNA nuclear export to inhibit host gene expression (Yarbrough et al., 2014). Most RNA viruses synthesize their mRNAs in the cytoplasm and do not depend on cellular mRNA nuclear export mechanism. In contrast, DNA viruses, including herpesviruses, risk hindering the exit of their own mRNAs if they block mRNA nuclear export. Nevertheless, SOX induces mRNA hyperadenylations and their nuclear retention due to global mRNA degradation in the cytoplasm (Kumar and Glaunsinger, 2010). Moreover, we identified KSHV ORF10 as another inhibitor of host gene expression by interacting with a cellular mRNA export factor, Rae1. We review the current knowledge regarding the molecular mechanisms of SOX and ORF10 as well as their roles in the context of viral lytic replication and pathogenesis (**Figure 1**).

## ORF37: A Viral Scissor for mRNA

Herpesviruses undermine mRNA stability through proteins that possess an endoribonuclease activity (Read, 2013). These include the virion host shutoff (vhs) protein encoded by HSV-1 UL41 (Fenwick and McMenamin, 1984; Strom and Frenkel, 1987; Elgadi et al., 1999) and KSHV SOX (Glaunsinger and Ganem, 2004). Unlike vhs, SOX originates from the herpesviral alkaline exonuclease protein family involved in viral DNA genome processing. Only SOX homologs of the gamma subfamily evolved a separate endonuclease activity causing host shutoff (Glaunsinger and Ganem, 2004; Rowe et al., 2007; Covarrubias et al., 2009). The alkaline exonuclease and host shutoff functions of SOX are genetically separable with specific mutations affecting only one of the two functions (Glaunsinger et al., 2005).

SOX causes host shutoff through a sequential action with a cellular 5’-3’ exonuclease, Xrn1 (PACMAN), resulting in cytoplasmic transcript degradation (Covarrubias et al., 2011). The current working model is that SOX internally cleaves the target transcripts in a non-random manner with preferred sites defined by a degenerate sequence motif (Gaglia et al., 2015; Mendez et al., 2018), providing Xrn1 with an entry point to complete degradation. In addition to site preferences, co-sedimentation of SOX with 40S ribosomal subunits indicates that SOX targets transcripts early during translation, further corroborated by SOX’s inability to initiate degradation of translation-incompetent reporters or RNA Pol I and Pol III transcripts (Covarrubias et al., 2011).

Remarkably, SOX targets both cellular and viral mRNAs (Abernathy et al., 2014). The MHV-68 SOX homolog shares the same host shutoff activity as KSHV SOX (Covarrubias et al., 2009). When the shutoff function of SOX is disabled in MHV-68, the majority of MHV-68 genes have higher mRNA levels (Richner et al., 2011). Not all MHV-68 genes with increased transcript levels have higher protein expression. However, this altered viral protein expression unexpectedly impacts the composition of virions produced by the SOX mutant without changing the ratios of particles to plaque forming units. In addition, the resulting changed virion composition affected viral entry. Despite enhanced viral gene expression, the SOX MHV-68 mutant is attenuated *in vitro* and *in vivo*. This is a surprising finding since host shutoff is generally thought to help viruses seize cellular resources to maximize their own protein expression for the highest virion production. Instead, SOX controls the viral gene expression to produce virions with a proper composition for optimal viral replication.

## ORF37: A Cytoplasmic Terminator With a Nuclear Impact on mRNA Export

As many RNA-binding proteins are capable of nucleo-cytoplasmic shuttling, a SOX-mediated massive decrease in cytoplasmic mRNA can lead to the nuclear relocation of cytoplasmic RNA-binding proteins (Gilbertson et al., 2018). PABPC, a predominantly cytoplasmic poly(A) binding protein, is one such nucleo-cytoplasmic shuttling protein (Afonina et al., 1998). It interacts with the cap-binding complex, eIF4F, through subunit eIF4G, to form a closed loop structure (Tarun and Sachs, 1996; Wells et al., 1998), which promotes the recruitment of ribosome 40S subunit for translation. Upon RNA binding, the PABPC nuclear import signal is masked, and thus when cytoplasmic polyadenylated transcripts drop substantially due to SOX activity, RNA-free PABPC is imported into the nucleus (Kumar et al., 2011). A SOX mutant that cannot accelerate mRNA turnover also fails in PABPC1 nuclear relocalization (Lee and Glaunsinger, 2009). Moreover, overexpression of a cytoplasmic deadenylase to reduce the abundance of cytoplasmic poly(A) RNA also induces nuclear import of PABPC1 (Lee and Glaunsinger, 2009). PABPC nuclear relocalization by SOX is observed during KSHV lytic replication (Lee and Glaunsinger, 2009). HSV-1 vhs can also cause nuclear accumulation of PABPC1 (Kumar and Glaunsinger, 2010), indicating a common impact in cytoplasmic mRNA drop. The major consequences of nuclear relocalization of PABPC1, by SOX, vhs, or the fusion with a nuclear retention signal (NRS) to PABPC1, are hyperadenylation of mRNAs and inhibition on their nuclear export (Kumar and Glaunsinger, 2010). Notably, nuclear PABPC1-NRS does not destabilize GFP reporter mRNA but diminishes GFP protein expression (Kumar and Glaunsinger, 2010), perhaps by retaining the GFP transcript in the nucleus. This indicates that in addition to cytoplasmic mRNA degradation, the impact of ORF37 on nuclear export of mRNA due to PABPC nuclear translocation is a critical part of its host shutoff function.

Nuclear mRNA hyperadenylation has been observed when RNAs are not exported (Hilleren and Parker, 2001; Jensen et al., 2001; Hammell et al., 2002; Qu et al., 2009; Bresson and Conrad, 2013). Since hyperadenylation and nuclear export are intimately linked, it is difficult to determine whether a block on export or hyperadenylation occurs first upon the PABPC nuclear relocation. SOX-induced hyperadenylation depends on PABPC1 and poly(A) polymerase, PAPII (PAPα) (Lee and Glaunsinger, 2009). The involvement of cleavage/polyadenylation CPA machinery is assumed. CPA is carried out by a large complex consisting of multi-subunit of cleavage and polyadenylation specificity factor (CPSF) and PAP that adds adenosines to the cleavage fragment (Proudfoot, 1996). Subsequently, nuclear poly(A)-binding protein (PABPN) associates with the newly-added poly(A) tail and stimulates PAPII to produce a long poly(A) tail of ~250 nucleotides (Bienroth et al., 1993; Kühn et al., 2009; Kühn et al., 2017). It has been proposed that additional adenosines beyond 250 residues cannot support a productive CPSF-PAPII complex for efficient polyadenylation (Wahle, 1995; Kühn et al., 2009). PABPN and CPSF contribute to the recruitment of nuclear export complex to mRNA (Shi et al., 2017). The loss of PABPN can lead to a shortened poly(A) tail and mRNA nuclear retention (Apponi et al., 2010). It is possible that relocating nuclear PABPC1 replaces PABPN on the poly(A) tail of mRNAs and prevents RNA export factor recruitment, interrupting mRNA nuclear export and causing subsequent hyperadenylation.

Despite a predominant nuclear SOX presence, cytoplasmic SOX largely mediates the effects of accelerated cytoplasmic mRNA decay, relocalization of PABC, and nuclear mRNA hyperadenylation (Covarrubias et al., 2009). SOX degrades cytoplasmic RNA, causing the PABPC1 nuclear import, which causes nuclear mRNA hyperadenylation and nuclear retention. Interestingly, the SOX impact on host gene expression in uninfected cells is less substantial compared to the extent of host shut-off during KSHV lytic replication (Clyde and Glaunsinger, 2011). This suggests that KSHV employs additional mechanisms beyond SOX to inhibit host gene expression, as other herpesviruses do (Rivas et al., 2016).

## ORF10: An Inhibitor in the mRNA Nuclear Exit

Mature mRNA are associated with a variety of proteins to form export-competent messenger ribonucleoprotein (mRNP) complexes (Carmody and Wente, 2009; Björk and Wieslander, 2017). Export of mRNPs requires the transit of these mRNPs through the nucleopore complex (NPC). An NPC consists of ~30 nucleoporins (Nups), with many containing phenylalanine-glycine (FG) repeats (Strambio-De-Castillia et al., 2010). At the central channel of nuclear pores, the FG-repeats of Nups form a permeability barrier (Terry and Wente, 2009). Majority of mRNA nuclear export through the NPCs involves the TAP-p15 (or NXF1-NXT1) heterodimer (Conti and Izaurralde, 2001; Köhler and Hurt, 2007; Carmody and Wente, 2009). The TAP-p15 heterodimers load mRNPs onto FG-containing Nups (FG-Nups) (Katahira et al., 1999; Bachi et al., 2000). The interaction of TAP with the FG repeats overcomes the permeability barrier of the nuclear pore, enabling mRNP translocation across the central channel (Grünwald et al., 2011; Powers and Forbes, 2012).

Our work identified a novel KSHV post-transcriptional regulator, encoded by ORF10, as an inhibitor of mRNA nuclear export (Gong et al., 2016). ORF10 expression leads to the reduction of cytoplasmic RNA levels for 24% of cellular genes, indicating a role of ORF10 in suppressing host gene expression during KSHV replication. While both ORF37 and ORF10 cause mRNA nuclear retention, ORF10 directly targets the nuclear export pathway by interacting with Rae1. We and others have independently identified, through mass spectrometry, that KSHV and MHV-68 ORF10 interacts with Rae1 (Davis et al., 2015; Gong et al., 2016), which is a highly conserved eukaryotic cellular export factor (Bharathi et al., 1997; Kraemer and Blobel, 1997; Sabri and Visa, 2000). Rae1 is involved in mRNA export by interacting with Nup98 at NPC (Pritchard et al., 1999; Blevins et al., 2003). We engineered a recombinant MHV-68 expressing FLAG-tagged ORF10 and identified Rae1 and Nup98 as ORF10-interacting proteins in the context of infection (Gong et al., 2016). Unlike TAP, Rae1 does not interact with the FG repeats of Nup98. Instead, the interaction is mediated through an evolutionarily conserved sequence within the Nup116/Nup98 family, referred to as Gle2-binding sequence (GLEBS) (Bailer et al., 1998; Pritchard et al., 1999). Rae1 also interacts with TAP (Bachi et al., 2000; Blevins et al., 2003) and thus, rather than functioning in the transit of mRNPs through NPCs, Rae1 potentially facilitates the docking of export-competent mRNPs onto NPCs. KSHV and MHV-68 ORF10s share 19% amino acid identity, highlighting the functional importance of Rae1 interaction during gammaherpesvirus infection. A structural study determined the MHV-68 ORF10 residues involved in the interactions with the Rae1-Nup98 complex (Feng et al., 2020). Some residues are highly conserved across the gammaherpesvirus ORF10 homologs with their mutations impairing interaction with the Rae1-Nup98 complex, causing the loss of mRNA nuclear export inhibition.

We have shown that KSHV and MHV-68 ORF10 induce nuclear accumulation of poly(A) RNA, which is abolished by mutations that disrupt ORF10-Rae1 interaction or Rae1 knockdown. Additionally, ORF10 is enriched at the nuclear rim. This localization depends on Rae1 and Nup98. Our current working model is that ORF10 interacts with Rae1, which in turn interacts with Nup98, to interfere with the Rae1-Nup98 complex function in mRNA export. While global mRNA export is not impacted by the absence of Rae1 (Babu et al., 2003), expression of the GLEBS domain of Nup98 induces nuclear accumulation of poly(A) RNA (Pritchard et al., 1999). The GLEBS domain sequesters Rae1 from binding to the wild type Nup98 at NPCs. ORF10 does not disrupt the interaction between Rae1 and Nup98; instead, it undermines the function of the complex. Both studies support a role of Rae1 in mRNA export, but the precise mechanism remains unknown. Cell fractionation combined with RNA sequencing indicates that the ORF10 impact on mRNA export is not global. While a strong correlation was found between RNA abundance and SOX-mediated degradation (Gilbertson et al., 2018), such correlation is not seen for ORF10-mediated export inhibition. It is possible that the Rae1-Nup98 complex is utilized by a subset of mRNAs for rapid exit of the nucleus. Without Rae1, this subset of mRNAs can still exit the nucleus albeit less efficiently, which can only be observed with careful kinetics studies.

Unlike SOX, we did not find viral transcript inhibition by ORF10. Herpesviral lytic genes are expressed in a cascade order and classified as immediate early, early and late genes. RNA sequencing showed that in the absence of ORF10, the transcription of late genes during KSHV lytic replication was most impacted, resulting in reduced virion production (Gong et al., 2016). This phenotype was recapitulated by the null function ORF10 mutant lacking Rae1 interaction or by Rae1 expression knockdown during KSHV lytic replication. These results indicate that ORF10 facilitates efficient viral late gene expression through its Rae1 interaction. Nevertheless, how the inhibition on nuclear mRNA export by ORF10 affects viral gene transcription requires further investigations.

## Rae1-Nup98: A Popular Viral Target

Gammaherpesviral ORF10 is not the only viral protein targeting Rae1-Nup98. Vesicular stomatitis virus (VSV) and influenza A virus (IAV) encode proteins that interact with Rae1 and inhibit mRNA export (Faria et al., 2005; Satterly et al., 2007), underscoring the importance of Rae1-Nup98 in viral replication. Recently, ORF6 encoded by severe acute respiratory syndrome coronavirus-2 (SARS-CoV-2) was found to interact with Rae1 (Gordon et al., 2020; Li et al., 2020), although the functional consequence is unknown (Miorin et al., 2020). A structural study suggests that the matrix (M) protein of VSV prevents Rae1 from binding to the RNA phosphate backbone, blocking Rae1 function in mRNA export (Quan et al., 2014). MHV-68 ORF10 also binds to a similar interface between Rae1-Nup98 as VSV M (Feng et al., 2020), highlighting a vulnerable structural aspect for viral exploitation. The C-terminal tail of MHV-68 ORF10 interacts with the RNA-binding groove of Rae1-Nup98 but on a different side from the M protein target. The binding of VSV M or gammaherpesviral ORF10 is expected to disrupt the RNA-binding ability of Rae1-Nup98. This prediction is true for the M-Rae1-Nup98 complex but not the ORF10-Rae1-Nup98 complex (Feng et al., 2020). It appears that ORF10 provides an alternative RNA-binding surface for the ORF10-Rae1-Nup98 complex. The IAV NS1 protein forms a complex with Rae1 and Tap-p15 heterodimer (Satterly et al., 2007) but currently no structural information is available on its interaction with Rae1. Embryonic fibroblasts from heterozygous knockout mice of Rae1 (Rae^-/+^) or Nup98 (Nup98^-/+^) or both (Rae^-/+^Nup98^-/+^) are more susceptible to IAV-induced cell death but produced more virions than the wild type cells (Satterly et al., 2007). This differs from our Rae1 knockdown results that show Rae1 requirement for efficient KSHV lytic replication (Gong et al., 2016). The likely explanation is that nuclear export of cellular genes is impacted differently by the Rae1-Nup98 complex with a subset of genes more dependent than others. mRNAs of several immune-related genes have higher nuclear to cytoplasmic ratios in Nup98^-/+^ and Rae^-/+^Nup98^-/+^ cells. Moreover, Nup98 is upregulated by interferons, and treatment by interferons can overcome VSV M-mediated inhibition on mRNA export (Enninga et al., 2002). Certain cellular genes may require the Rae1-Nup98 complex for exporting their mRNAs into the cytoplasm. Some of these Rae1-dependent genes may encode antiviral proteins, which accounts for the increased replication of IAV in the absence of fully functional Rae1-Nup98. However, there could also be cellular mRNAs that do not need Rae1 for export but recruit it for rapid export under special conditions, such as stress (Izawa et al., 2004). Through Rae1 interaction, ORF10 gains access to Rae1-regulated mRNAs and inhibits their export to promote viral replication.

## Concluding Remarks

KSHV encodes SOX and ORF10, known to inhibit host gene expression through distinct molecular mechanisms. SOX targets viral transcript for degradation, resulting in reduced viral protein expression, maintaining virion production with balanced composition (Abernathy et al., 2014). In contrast, ORF10 does not seem to impact nuclear export of viral mRNAs but is required for efficient expression of viral proteins and virion production (Gong et al., 2016). While SOX and ORF10 are capable of inhibiting host gene expression, their functions during gammaherpesvirus infection do not overlap. Moreover, SOX and ORF10 have different timings of expression during KSHV lytic replication; the former is an early gene and the latter is a late gene (Arias et al., 2014). Does ORF37 coordinate with ORF10 to down-regulate host gene expression for optimal viral replication or does their inhibition on mRNA export serve different purposes? Investigating the roles of ORF10 and ORF37 in the context of infection combined with cell fractionation and RNA sequencing will provide insight into their impact on host gene expression. Additionally, the impact of these two viral genes on the host proteome remained to be determined. Due to the functional conservation of ORF10 and ORF37 in KSHV and MHV-68, a combination of *in vitro* molecular biology and *in vivo* infection model with MHV-68 will certainly provide a comprehensive overview of their functions in viral pathogenesis. ORF10 interacts with Rae1 to achieve export inhibition, yet Rae1 functions in RNA export is still largely unclear. Therefore, ORF10 also serves as a valuable tool to understand this cellular pathway that is targeted by multiple viruses.

## Author Contributions

CP and T-TW wrote the manuscript and prepared the figure. All authors contributed to the article and approved the submitted version.

## Funding

This work was funded by NIH National Institute of Dental and Craniofacial Research, grant number R01DE028774.

## Conflict of Interest

The authors declare that the research was conducted in the absence of any commercial or financial relationships that could be construed as a potential conflict of interest.
